# Recoverin depletion accelerates cone photoresponse recovery

**DOI:** 10.1098/rsob.150086

**Published:** 2015-08-05

**Authors:** Jingjing Zang, Jennifer Keim, Edda Kastenhuber, Matthias Gesemann, Stephan C. F. Neuhauss

**Affiliations:** Institute of Molecular Life Sciences, Neuroscience Center Zurich and Center for Integrative Human Physiology, University of Zurich, Winterthurerstrasse 190, Zurich 8057, Switzerland

**Keywords:** phototransduction termination, zebrafish, cone photoreceptor, electroretinogram

## Abstract

The neuronal Ca^2+^-binding protein Recoverin has been shown to regulate phototransduction termination in mammalian rods. Here we identify four *recoverin* genes in the zebrafish genome, *rcv1a*, *rcv1b*, *rcv2a* and *rcv2b*, and investigate their role in modulating the cone phototransduction cascade. While Recoverin-1b is only found in the adult retina, the other Recoverins are expressed throughout development in all four cone types, except Recoverin-1a, which is expressed only in rods and UV cones. Applying a double flash electroretinogram (ERG) paradigm, downregulation of Recoverin-2a or 2b accelerates cone photoresponse recovery, albeit at different light intensities. Exclusive recording from UV cones via spectral ERG reveals that knockdown of Recoverin-1a alone has no effect, but Recoverin-1a/2a double-knockdowns showed an even shorter recovery time than Recoverin-2a-deficient larvae. We also showed that UV cone photoresponse kinetics depend on Recoverin-2a function via cone-specific kinase Grk7a. This is the first *in vivo* study demonstrating that cone opsin deactivation kinetics determine overall photoresponse shut off kinetics.

## 1. Introduction

The vertebrate retina contains two classes of photoreceptors, rods and cones, which function at low and bright light conditions, respectively. Although both share a similar G-protein-coupled phototransduction pathway, the cone photoresponse is characterized by lower sensitivity and faster kinetics, which allows cones to function over almost 9 orders of illumination magnitude [1]. The rate-limiting step of photoresponse recovery is also different between these two cell types [2–4]. Rods and cones use cell-type-specific molecules in the phototransduction cascade, which may account for these differences.

The cascade is initiated by light-activated rhodopsin (Rh*), which induces the detachment of trimeric G-protein Transducin *α*-subunit. The Transducin *α*-subunit in turn binds to phosphodiesterase (PDE), causing a decrease in the second messenger cGMP. This drop in cGMP levels leads to the closure of CNG cation channels, hyperpolarizing the photoreceptor and lowering [Ca^2+^]_i_ [5]. The deactivation of both Rh* and the PDE–Transducin complex is required to terminate the phototransduction cascade. Rh* in rods is initially phosphorylated by Rhodopsin kinase Grk1 [6] in a Ca^2+^-dependent manner via the small Ca^2+^-binding protein Recoverin [7,8]. Rod Recoverin is proposed to inhibit Grk1 in darkness when outer segment [Ca^2+^]_i_ is high and is released from Grk1 during light response when [Ca^2+^]_i_ is decreasing [9,10]. Modulation of Rhodopsin lifetime during light adaption is abolished in Recoverin-deficient mice [11]. The final deactivation of Rh* is achieved by the binding of Arrestin [12], while intrinsic GTPase activity ends the ability of PDE–Transducin complex to hydrolyse cGMP [13].

Cone-specific opsin kinase Grk7 has similar functions as rod Grk1 [14,15]. Interestingly, cone Arrestin is not essential to deactivate short-wavelength opsin in mice [16,17], but its functional loss induces delayed cone photoresponse recovery in cone-dominant zebrafish [18]. A Ca^2+^-sensitive phosphorylation of cone visual pigment *in vivo* in zebrafish [19] indicates a possible role of Recoverin in regulating cone opsin quenching via direct control of [Ca^2+^]_i_ during light response.

In this study, we take advantage of the cone-dominant retina of zebrafish to study Recoverin function in cone vision. The zebrafish genome harbours four *rcv* genes. While Recoverin-1b (Rcv1b) is only expressed in the adult retina the other Recoverins are present throughout development in all cone types, with the exception of Rcv1a, which is expressed in rods and UV cones only.

We determined photoresponse recovery with a double flash electroretinogram (ERG) paradigm [14] analysing the role of Recoverin proteins. Our results establish that the cone photoresponse kinetics are shaped by photopigment quenching and a loss of Recoverin leads to an accelerated photoresponse recovery.

## 2. Material and methods

### 2.1. Zebrafish care

Zebrafish (*Danio rerio*) were kept at a 14 L : 10 D cycle at 28°C [20]. Embryos of WIK wild-type fish were raised in E3 medium containing 0.01% methylene blue or with 0.2 mM PTU (1-phenyl-2-thiourea; Sigma-Aldrich) to avoid pigmentation. Adult zebrafish were sacrificed by fish system water containing 0.4% 3-aminobenzoic acid methyl ester (MESAB, Sigma-Aldrich) and 4.6 mM NaHCO_3._

### 2.2. Cloning of *recoverin* genes and *in situ* hybridization

Cloning was performed as described in [21] using oligonucleotide primers listed in electronic supplementary material S1. Digoxigenin-labelled *in situ* hybridization (ISH) RNA probes were generated according to supplier's instructions (DIG RNA Labelling Mix, Roche) and used on zebrafish larvae as previously described [22].

### 2.3. Generation of antibodies

Custom polyclonal peptide antibodies were raised by Eurogentec (Seraing, Belgium). Rabbits were immunized with the Rcv1a peptide CIQYDEPKKIQEKLKEKKH or Rcv2b peptide CKLIPKDKQTSLPNDES. Guinea pigs were immunized with the Rcv1b peptide CIQFDKPQKVQEKLKEKTQ or Rcv2a peptide CKLIPKEDQESLPADEN. Antibodies were affinity-purified.

### 2.4. Immunohistochemistry

Section immunohistochemistry was carried out as described previously [14], except for the following modifications. Adult zebrafish eyes were fixed in 2% trichloroacetic acid (Sigma-Aldrich, Switzerland) for 30 min at room temperature. Primary Recoverin antibodies were diluted 1 : 400. Chicken anti-GFP antibody (Sigma-Aldrich, dilution 1 : 500) and mouse anti-PCKalpha (MC5) antibody (Novus Biologicals, NB200-586; 1 : 500) were used. All the secondary antibodies (Invitrogen) were diluted 1 : 1000 in PBS.

Whole-mount immunohistochemistry was performed on 5 days post-fertilization (dpf) larvae. The larvae were fixed in 2% tricholoroacetic acid at 4°C for 30 min, dehydrated in a methanol series and stored in 100% methanol for at least 24 h, then incubated in ice-cold acetone for 7 min before blocking.

### 2.5. Microscopy

ISH images were taken by a light microscope (BX61, Olympus) with a CCD camera (ColorView IIIu, Soft Imaging System, Olympus) and processed by Adobe Photoshop CS3. Fluorescence Z-stacks photos were taken by a confocal laser scanning microscope (Leica SP5, Leica Microsystems) and processed by Imaris 7.6.3 (Bitplane, Zurich, Switzerland).

### 2.6. Targeted gene knockdown

Antisense morpholino oligonucleotides (Gene Tools, Philomath, OR, USA) were designed against translational start sides and injected into one cell-stage embryos (electronic supplementary material S1).

Amounts of morpholino per embryo were *rcv1a* (20 ng), *rcv2a* (7.4 ng or 3.6 ng), *grk7a* (2.4 ng), *rcv2b* (1.2 ng) and control (7.4 ng). For double knockdowns, *rcv2a* (3.6 ng)/*grk7a* (2.4 ng) or *rcv1a* (11 ng)/*rcv2a* (7.4 ng) were injected.

### 2.7. Western blot

Twenty to forty 5 dpf larvae were homogenized in 150 µl RIPA buffer (150 mM NaCl, 1% Triton-X, 0.5% sodium deoxycholate, 50 mM Tris (pH 8), 1 mM EDTA, 0.1% SDS). *β*-Actin (approx. 42 kDa) was used as a loading control. Primary antibodies were diluted to the following concentrations: Rcv1a: 1 : 1,000; Rcv2a: 1 : 2000; Rcv2b: 1 : 1000 *β*-Actin: 1 : 3000. Secondary horseradish peroxidase (HPR)-linked antibodies (Invitrogen) were diluted in blocking buffer (goat anti-rabbit: 1 : 15 000; rabbit anti-guinea pig: 1 : 25 000; goat anti-mouse: 1 : 10 000). Signal was detected by the LAS 4000 Chemiluminescence Imager (software: Image Quant LAS 4000, automatic exposure) and knockdown levels were semi-quantified by ImageJ [23].

### 2.8. Normal and spectrum electroretinography

Normal (white) ERG was recorded as previously described [24]. Full light intensity (light source: Zeiss XBO 75 W) was measured by spectrometer (Ocean Optics, USB2000+) with spectrum shown in S2A (SpectraSuite, Ocean Optics). For the 100% double flash paradigm, pairs of two light flashes with the same intensity and duration (500 ms) were given [14]. The interval between two flashes was increasing (100, 200, 300, 500, 1000, 2000, 3500 and 5000 ms). A neutral density filter was applied to have 0.1% double white flash paradigm and the interval between two flashes was progressively increasing (100, 150, 200, 250, 300, 350, 400, 450 and 500 ms). The interval between two pairs was always 10 s for both bright light and dim light response. Spectrum ERG used the similar set-up but additional background light source (Philips projection lamp type 6958, 20 V, 250 W; housing: Liesegang Diafant 250) with a short wavelength absorbing filter and an UV light filter in front of white light source (electronic supplementary material S2B). After calibration, the spectrum graph showed that most of the light above 385 nm in the visible range was blocked. There was indeed a peak at around 720 nm. To make sure this peak cannot generate any electrical response in the retina, another short wavelength absorbing filter was applied together with this UV light filter to receive only the light at around 720 nm. But even in darkness this light did not give any ERG response. The background light was used to adapt the blue, green and red cones with minimal activation for UV cones. For paired UV flash recordings, flash interval and duration were the same as for dim flash recordings with one extra interval of 750 ms. All the experiments were performed at room temperature (22°C).

## 3. Results

In order to explore the role of Recoverins (Rcvs) in the termination of the visual transduction cascade, we cloned four zebrafish *recoverin* (*rcv*) orthologues, namely *rcv1a, rcv1b, rcv2a* and *rcv2b*.

### 3.1. Recoverins are expressed in photosensitive organs

**Figure 1.**
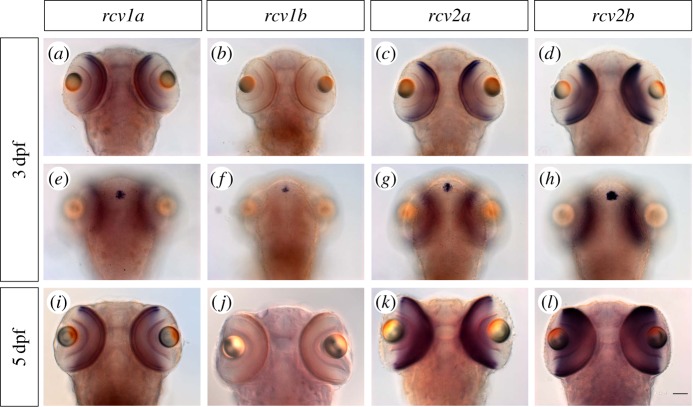
Expression of *rcv* genes in 3 dpf and 5 dpf zebrafish larvae. (*a–h*) All the *rcv* genes except *rcv1b* showed expression in 3 dpf larvae retina. All the *rcv* genes were expressed in the pineal gland. (*i–l*) *rcv1b* still showed no expression in the 5 dpf larval retina. Scale bar (=50 µm) applies to all panels.

We determined both the RNA and protein expression of the Recoverins in larval and adult zebrafish. All four *rcvs* transcripts are expressed in the pineal gland, a photosensitive organ, starting at around 2 dpf. At around 3 dpf, expression is initiated in the ventral outer retina, with the exception of *rcv1b* transcripts, which are absent from the larval retina (figure 1). At 5 dpf, when the retina becomes fully functional with the exception of rods that do not significantly contribute to visual function at this stage [25], *rcv1a, rcv2a* and *rcv2b* transcripts are expressed throughout the photoreceptor cell layer.

The zebrafish retina contains one rod and four cone types, namely UV-sensitive (expressing the *opn1sw1* opsin gene) and blue-sensitive (*opn1sw2*) short single cones and double cones that have a red- (*opn1lw*) and green- (*opn1mw*) sensitive member [26].

**Figure 2.**
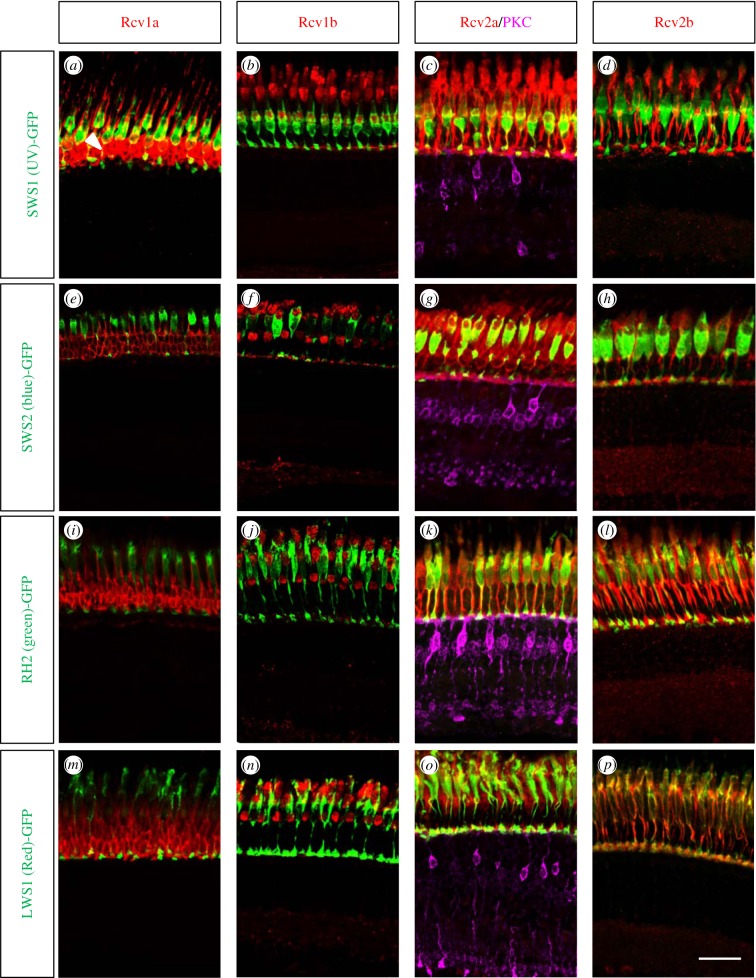
Zebrafish Recoverins are expressed in the different cone subtypes. Adult retinal sections from transgenic zebrafish highlighting the different cone subtypes were co-stained with Rcv antibodies. White arrowhead in (*a*) marked the rod photoreceptors. While Rcv1b, Rcv2a and Rcv2b proteins are present in all cone subtypes, Rcv1a is only expressed in rods and UV cones. In order to highlight also ON-bipolar cells, Rcv2a antibodies were supplemented with PKC antibodies (violet staining). Scale bar (=20 µm) applies to all panels.

In order to determine the cellular and subcellular localization of the Rcv proteins, we generated paralogue-specific antibodies against all four Rcvs and performed immunohistochemistry on adult retinal section of different transgenic lines with GFP-labelled cone subtypes [27–30]. We detected Rcv1a expression in rods and UV cones (figure 2), while the two Rcv2 paralogues were expressed in all cone subtypes. Rcv2a also showed staining in the inner nuclear layer at the level of bipolar cells (electronic supplementary material S3), where the labelling partially colocalized with the ON-bipolar cell marker protein kinase C alpha (PKC). Interestingly, this inner retinal staining was neither detected by ISH (figure 1) nor immunohistochemical staining in the larval retina (electronic supplementary material S4A). Rcv1b protein was found in all adult cone photoreceptors (figure 2), while being absent in the larval retina (figure 1).

### 3.2. UV cone photoresponse recovery is accelerated in Rcv-deficient zebrafish larvae

To study the function of Recoverin proteins, we designed morpholinos against *rcvs* and cone-specific opsin kinase Grk7 [14]. The knockdown efficiency at 5 dpf was evaluated by Western blot and whole-mount immunohistochemistry (electronic supplementary material S4). The levels of each Rcv protein were largely reduced when it was knocked down while levels of the other Rcv proteins were maintained, indicative of both knockdown efficacy and antibody specificity. The knockdown efficiency was in the range of 90% (Rcv1a, 93%; Rcv2a, 97%; Rcv2b, 88%), as semi-quantified by Western blots using *β*-actin as loading control (electronic supplementary material S4B).

In order to study the function of the only mammalian orthologue Rcv1a in larvae and to investigate how different Recoverins modulate opsin lifetime in the same cell, we used spectral ERG to isolate photoresponse, which was largely contributed by UV cones. At 5 dpf, photoresponses are considered to be purely cone driven [25]. Additionally, UV cones can be selectively stimulated by UV light with minimal activation of the other cone subtypes.

**Figure 3.**
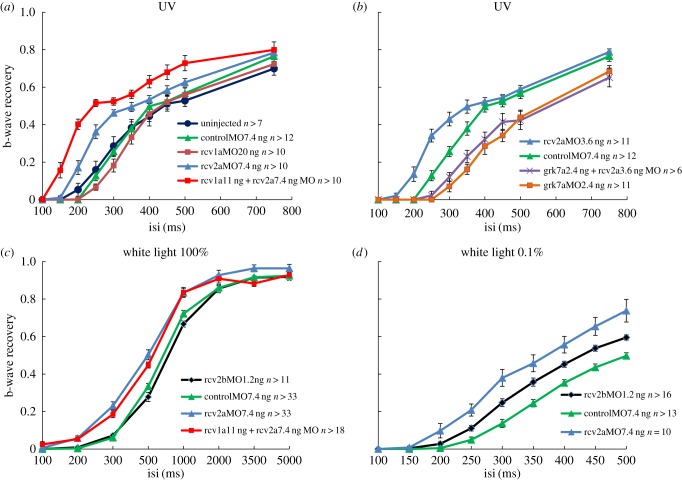
Cone photoresponse recovery is accelerated in Rcv-deficient larvae. Time course of b-wave recovery in (*a,b*) UV spectral ERG, (*c*) maximum white light ERG and (*d*) dim white light ERG are shown. Under UV light conditions recoverin2a and combination of recoverin1a and recoverin2a are effective in accelerating the response recovery (*a*), whereas depleting the effort kinase Grk7a as expected prolongs the recovery time (*b*). Note that Rcv2a knockdown accelerates response recovery under normal and dim white light conditions, whereas Rcv2b only accelerates recovery under dim light conditions (*c,d*). Data are presented as mean ± s.e.m.

In the double flash paradigm [14], uninjected WT larvae showed similar b-wave recovery compared with control morphants (*p* > 0.05; figure 3*a*). Although there was less than 10% protein left in Rcv1a morphants, they did not show any significant acceleration of photoresponse recovery (*p* > 0.05). When Rcv2a was knocked down (figure 3*a*), b-wave recovery was accelerated (*p* < 0.005 at interval 200–300 ms). Double knockdown of Rcv1a and Rcv2a further accelerated the recovery time (*p* < 0.005 at interval 150 ms and 200 ms compared with Rcv2a single knockdown). In order to recover to 40% of their dark levels, Rcv1a and Rcv2a double knockdown, Rcv2a single knockdown and control morphants required around 200 ms, 275 ms and 350 ms, respectively. Rcv1a knockdown did not induce any phenotype, but Rcv1a and Rcv2a double defect larvae showed significant acceleration compared with Rcv2a single knockdown, indicating that the function of Rcv1a can be replaced by Rcv2a. Concomitant reduction of Rcv2a and Rcv1a cannot be compensated any more. The fact that recovery was faster rather than slower in morphants argues against a toxic side effect of morpholino injection.

Because Rcv proteins are highly expressed in the photoreceptor synaptic terminal (figure 2; electronic supplementary material, figure S4) and Rcv1 in mice has been reported to modulate synaptic transmission in the rod pathway [31], it is possible that the faster b-wave recovery in the morphants not only comes from the faster phototransduction decay, but is additionally mediated by an effect on synaptic transmission. The contribution of Rcv downregulation to synaptic transmission can be easily evaluated by quantifying the b-wave amplitude and time-to-peak (table 1). Indeed in this experiment, we did not find any difference between morphants and controls, arguing that synaptic transmission is not affected, which suggested the effect we observed in b-wave recovery is purely contributed by the accelerated phototransduction termination.

**Table 1.** Amplitude and time course of ERG b-wave in Rcv-deficient larvae is not significantly different with control. All the data are shown as mean ± s.e.m. One-way ANOVA shows *p* > 0.05 in all cases.

	UV				
	control	Rcv2aMO	Rcv1aMO	Rcv1a & 2aMO	
amplitude (µV)	92.6 ± 20.1	130.4 ± 13.4	96.5 ± 14.3	85.3 ± 14.1	
*T*_peak_ (ms)	249.3 ± 8.0	250.6 ± 7.9	253.6 ± 5.4	246.6 ± 5.9	

	100% white		0.1% white		
	control	Rcv2aMO	control	Rcv2aMO	Rcv2bMO
amplitude (µV)	716.0 ± 49.0	765.6 ± 72.0	182.4 ± 22.4	210.2 ± 51.8	149.9 ± 20.4
*T*_peak_ (ms)	206.0 ± 4.4	200.8 ± 6.0	243.2 ± 8.8	249.0 ± 8.6	253.0 ± 11.2

### 3.3. Recoverin affects SWS1 recovery via Grk7a

In rod photoreceptors, it is known that Recoverin regulates the lifetime of activated rhodopsin via Rhodopsin kinase [7]. In order to confirm that cone-specific visual pigment kinase Grk7a (electronic supplementary material S5) interacts with Recoverin, we compared the photoresponse recovery in Grk7a single knockdown larvae with the recovery time in double knockdowns of Grk7a and Rcv2a (figure 3*b*).

We confirmed that the response recovery is significantly delayed in the absence of Grk7a not only under normal ERG [14] but also in UV spectrum ERG (*p* < 0.05 at interval 250–500 ms), indicating Grk7a is a general cone opsin kinase (figure 3*b*). Importantly, the photoresponse recovery was not influenced by the additional reduction of Rcv2a (*p* > 0.5), indicating that Rcv2a acts via Grk7a. Hence, Recoverin regulates SWS1 opsin lifetime via cone opsin kinase.

### 3.4. Rcv2a and Rcv2b depletion accelerate photoresponse recovery under varying light conditions

It was suggested that white light ERG is dominated by the double cone photoresponse [18]. The fact that (unlike UV response) there was no significant difference in b-wave recovery between Rcv1a and Rcv2a double knockdown and Rcv2a single knockdown larvae in the double white flash paradigm proved this notion for the first time (figure 3*c*). Indeed, there was a significant acceleration of b-wave response recovery in *rcv2a* morphants with paired saturating flashes compared with control morphants (figure 3*c*). The *rcv2a* morphant recovery at an interval of 300 ms was three times faster than in control morphants. However, normal response kinetics were observed in *rcv2b* morphants (*p* > 0.1). Nevertheless, under dim flash conditions (0.1% of maximal light intensity) downregulation of *rcv2a* and *rcv2b* both accelerated the response decay (*p* < 0.05 at intervals from 250 ms to 500 ms; figure 3*d*). It took about 400 ms for *rcv2a* morphants, 450 ms for *rcv2b* morphants and 550 ms for the controls to recover about half of the photoresponse, suggesting a role of *rcv2b* in modulating the photoresponse kinetics only at a smaller Ca^2+^ dynamic range.

## 4. Discussion

Rhodopsin quenching requires the phosphorylation by rhodopsin kinase [6,32] and subsequent binding of Arrestin [33]. Rhodopsin kinase is regulated by Recoverin in a Ca^2+^-dependent manner [7,8]. Exogenous Recoverin prolongs the rod response [34] and genetic deletion does the opposite [35]. Before this study, little was known about the function of Recoverin in cones. But both the Ca^2+^-sensitive cone opsin phosphorylation in zebrafish [19] and the Ca^2+^-sensitive cone opsin quenching step dominating the overall response kinetics in salamander [3,4] imply an important role of Recoverin in shaping the cone photoresponse.

Four *recoverins* were found in the zebrafish genome. Larval zebrafish double cones exclusively express *rcv2a* and *rcv2b*. During bright light conditions, Rcv2a works to delay red and green opsin quenching, while both Rcv2a and Rcv2b work under dim light conditions. Hence Rcv2b contributes only when Ca^2+^ dynamic range is small [36,37] and less opsin is bleached, suggesting that Rcv2a may be the primary Recoverin and works over broader light conditions. This suggests variations in Ca^2+^ sensitivity among Recoverin isoforms, which would correlate with different numbers of lysine residues at the C-terminus of various Rcvs. The number of positively charged lysine residues indirectly determines the differences in membrane affinity and efficiency of rhodopsin kinase inhibition between salamander rod and cone Recoverin [38,39]. A reduced number of lysine residues results in decreased Ca^2+^ sensitivity and requires higher Ca^2+^ concentrations to inhibit rhodopsin kinase [40]. Rcv2a and Rcv2b possess 2 and 1 lysine residues at the C-terminus, respectively. Another consistent mechanism for Rcv2b working in dim light conditions could be different functional pairs of Grk and Recoverin. Two paralogous genes of *grk7* (*grk7a* and *grk7b*) and one paralogue for *grk1* (*grk1b*) were found to be expressed in zebrafish cones, while *grk1a* is restricted to rods [14,41]. The detailed cellular distribution of Grks in cones is not known, but Grk7a is clearly expressed in all cone subtypes (electronic supplementary material S5). According to Rcvs expression pattern and functional analysis, there are at least two functional pairs in zebrafish: Grk1a-Rcv1a exclusively in rods and Grk7a-Rcv2a in UV cones. The specific activity of Grk7a for rhodopsin phosphorylation is around 30 times higher than that of all the other Grks [41]. Rcv2b may regulate Grk1b or Grk7b instead of Grk7a, which would be consistent with Rcv2b working under different conditions than Rcv2a. Different Ca^2+^ sensitivity and Grk affinity may both contribute to different working ranges between Rcv2a and Rcv2b.

In rods, it is believed that Ca^2+^ regulates light response and light adaptation via three mechanisms: the dynamic drop in intracellular Ca^2+^ concentration accelerates rhodopsin phosphorylation [7], speeds up the synthesis of cGMP through guanylyl cyclase [42] and enhances the cGMP affinity of CNG-channels [43]. In cone photoreceptors there is a larger fraction of dark current carried by Ca^2+^ [44], a faster light-induced Ca^2+^ dynamic decline and a bigger Ca^2+^ dynamic range than in rods [37,45]. Furthermore, cones show lower sensitivity, faster response kinetics and adaption to a much wider range of light intensity than rods. It is expected that there is a more powerful Ca^2+^ negative feedback in cones. The Ca^2+^-dependent regulation on CNG channel ligand sensitivity is significantly more potent in cones with CNG-modulin as a modulator than in rods using calmodulin [46,47]. However, the Ca^2+^-sensitive guanylyl cyclase activating proteins (GCAPs) show weaker modulation in mammalian cones than in rods [48]. Ca^2+^ probably meditates a more powerful feedback via visual pigment phosphorylation in cones than in rods because the rate-limiting step of bright light response recovery in cones has been found to be opsin quenching [3,4], instead of Ca^2+^ insensitive PDE–Transducin deactivation in rods [2].

Our study is the first *in vivo* demonstration that cone opsin phosphorylation is indeed regulated by the Ca^2+^-binding protein Recoverin, which allows Ca^2+^ to directly control cone response kinetics and modulate the time scale during light response and adaptation.

## Supplementary Material

SUPPLEMENT LEGENDS.pdf

## Supplementary Material

S1.pdf

## Supplementary Material

Oligonucleotide Sequences

## Supplementary Material

Co-staining of Rcv2a and PKC antibodies on retina sections

## Supplementary Material

Morpholino knockdown of Rcv1a, Rcv2a, and Rcv2b in 5dpf Larvae

## Supplementary Material

Grk7a expression in adult retina sections

## Data accessibility

Genbank accession numbers: KT325590–KT325593.
