# Supplementary material for: Recoverin depletion accelerates cone photoresponse recovery
Source: Open Biol. 2015 Aug 5;5(8):150086. doi: 10.1098/rsob.150086 (PMC4554923; doi:10.1098/rsob.150086)
Supplement: SUPPLEMENT LEGENDS.pdf [file rsob150086supp1.pdf]

S1. Oligonucleotide Sequences

Sequences of primers and morpholino oligonucleotides in this study.

S2.Spectrum of ERG light

(A) Spectrum of full white light; (B) Spectrum of Spectrum ERG light: spectrum of background light with short wavelength filter is shown in red; spectrum of UV stimuli was shown in blue; spectrum of UV stimuli with short wavelength filter is shown in purple.

S3. Co-staining of Rcv2a and PKC Antibodies on Retina Sections.

Z-projections of confocal image stacks of immunochemical staining on adult retinas. White arrowhead marked the bipolar cell which was labeled by both Rcv2a and PKC antibodies. Yellow arrowhead marked the bipolar cell which was only labeled by Rcv2a antibody. Blue arrowhead marked the ON-bipolar cell which was only labeled by PKC antibody. Scale bar=20 µm.

S4. Morpholino Knockdown of Rcv1a, Rcv2a, *and* Rcv2b in 5 dpf Larvae.

(A)Whole-mount immunochemical staining of *rcv1a*, *rcv2a* and *rcv2b* morphants. Each *rcv* morphants were stained in the same well as control morphants. Scale bar = 30µm. (B) Knock down level was analysed by western blot. β-Actin was used as a loading control.

S5. Grk7a Expression in Adult Retina Sections.

Z-projections of confocal image stacks of immunochemical staining on adult retina. Grk7a showed overlapping expression with zpr-1 which labels double cones. White arrowhead marked long single (blue) cone outer segment and yellow arrowhead marked short single (UV) cone outer segment.
