# Supplementary material for: Recoverin depletion accelerates cone photoresponse recovery
Source: Open Biol. 2015 Aug 5;5(8):150086. doi: 10.1098/rsob.150086 (PMC4554923; doi:10.1098/rsob.150086)
Supplement: S1.pdf [file rsob150086supp2.pdf]

S1. Primer and Morpholino Sequences

rcv1a s GGACCAGAGTACAATTTAAG	as GAAGCTCTAATCAGTCATAG
rcv1b s CAGACCAGCACCACATAC	as TCTTGCACTTTCTGTGGTT
rcv2a s CAACATCTTTCTGAGCCC	as ATAGCGTCTTCATTCTCC
rcv2b s CACTCAGACAGAAGTCAT	as GTAGACCATCATCGCTTG

rcv1a 5’-CCATATTCCTTAAATTGTACTCTGG-3’
rcv2a 5’-GGCATTCCCCATTTTTGAAGGCTTG-3’
grk7a 5’-ATCGAGTCCCCCCATGTCACACATT-3‘
control 5’-CCTCTTACCTCAGTTACAATTTATA-3’
