# Supplementary figures and images for: Recoverin depletion accelerates cone photoresponse recovery
Source: Open Biol. 2015 Aug 5;5(8):150086. doi: 10.1098/rsob.150086 (PMC4554923; doi:10.1098/rsob.150086)

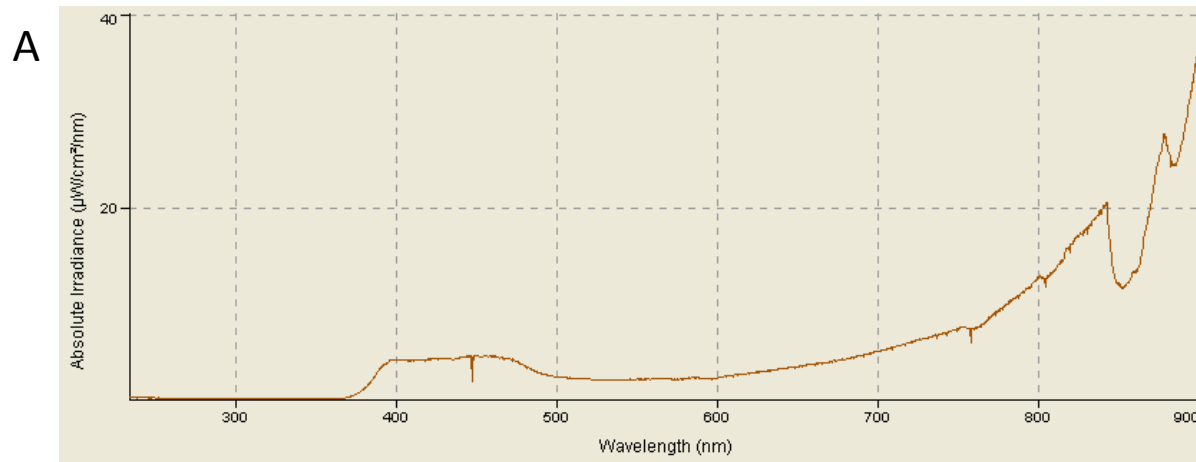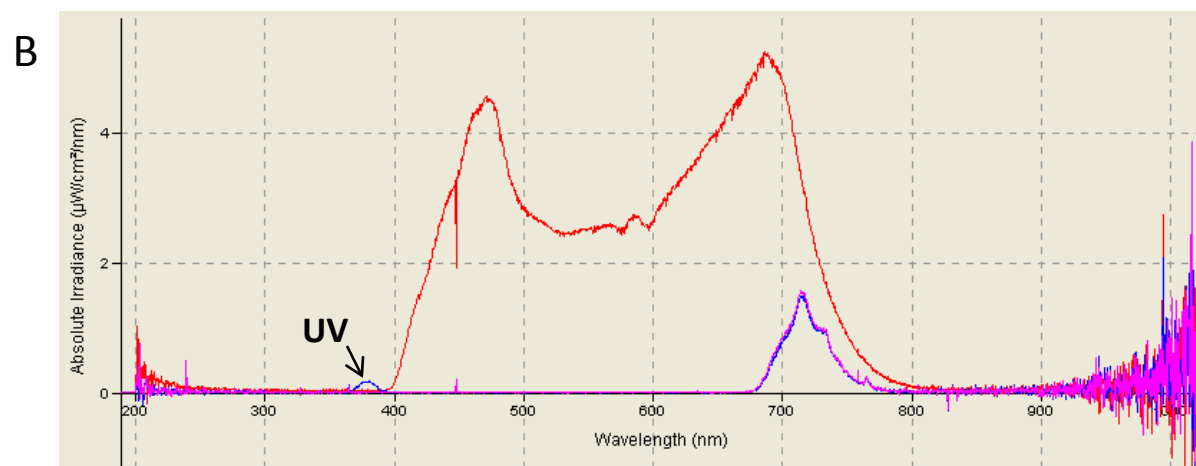

Supplement: Oligonucleotide Sequences [file rsob150086supp3.pdf]

Rcv2a

PKC $\alpha$ MC5

Rcv2a/PKC $\alpha$ MC5

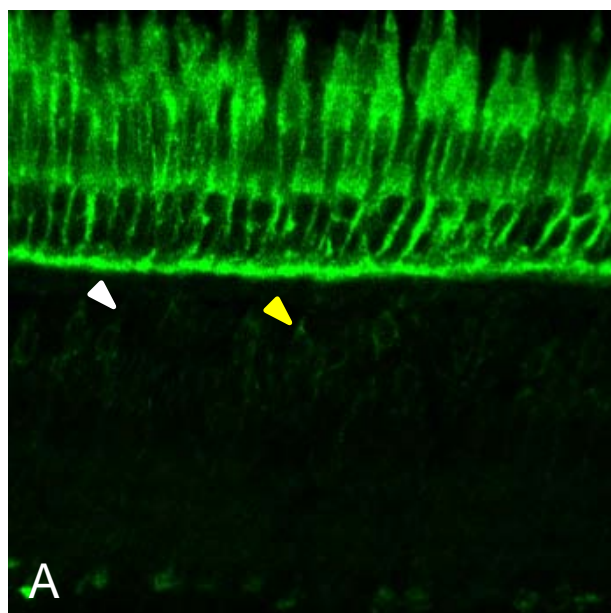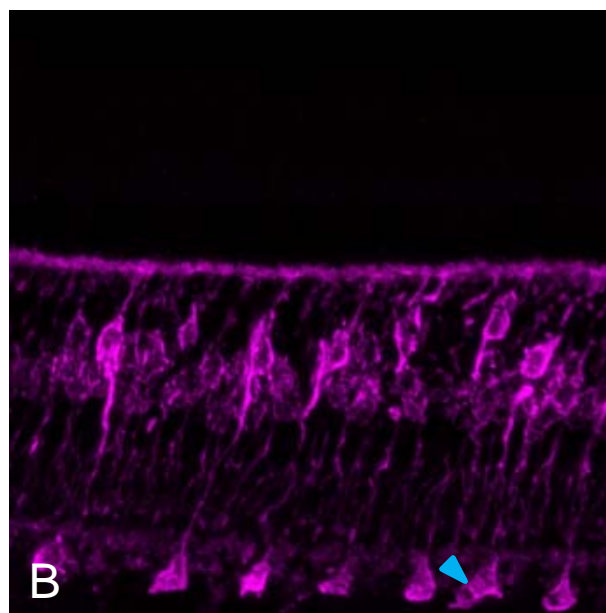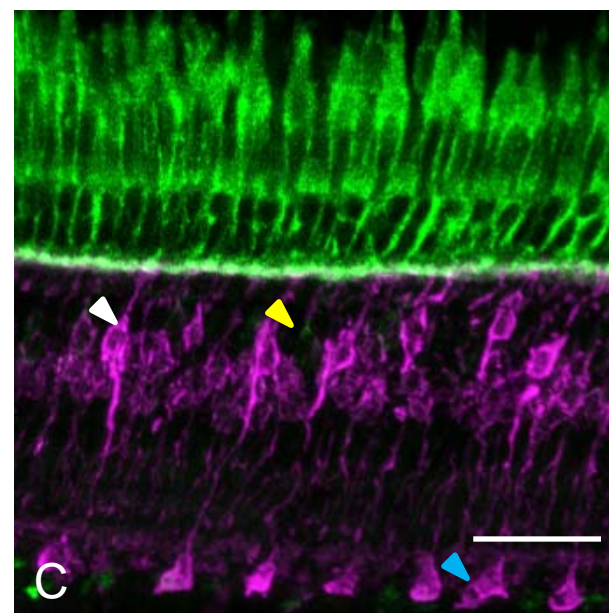

Supplement: Co-staining of Rcv2a and PKC antibodies on retina sections [file rsob150086supp4.pdf]

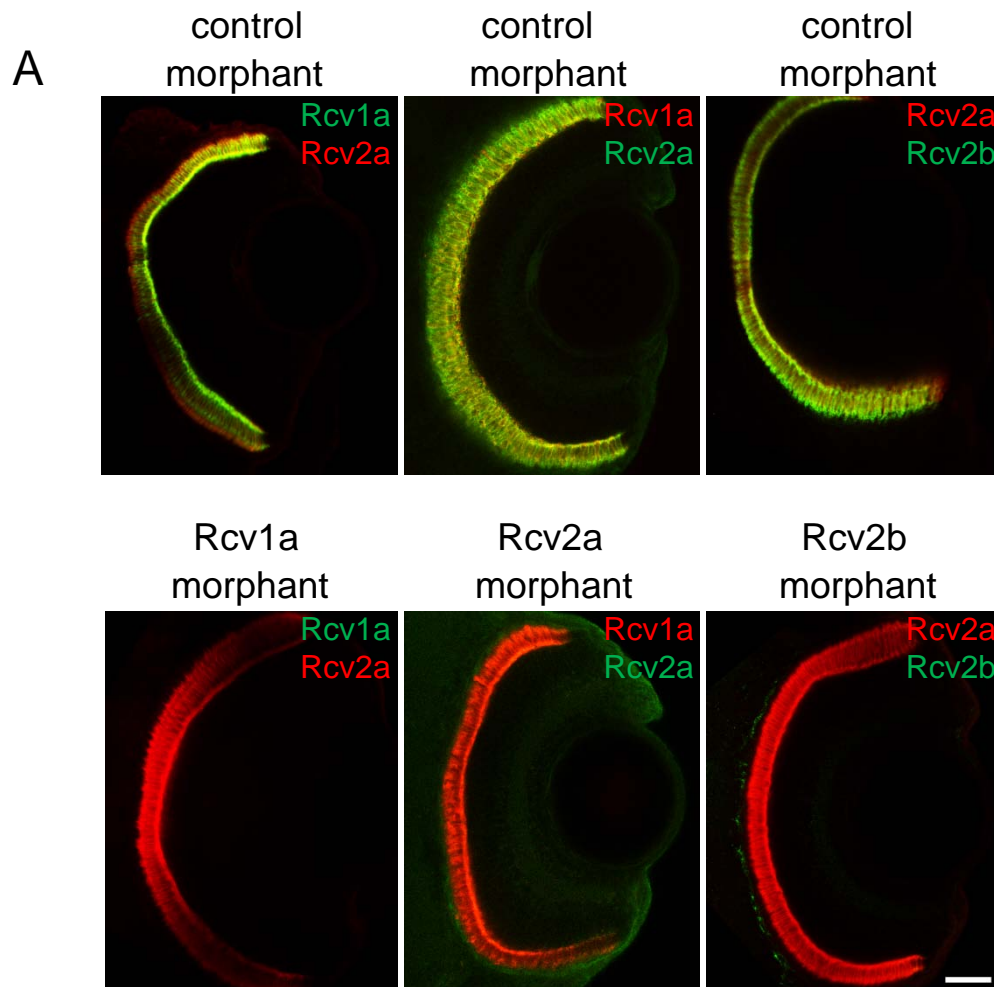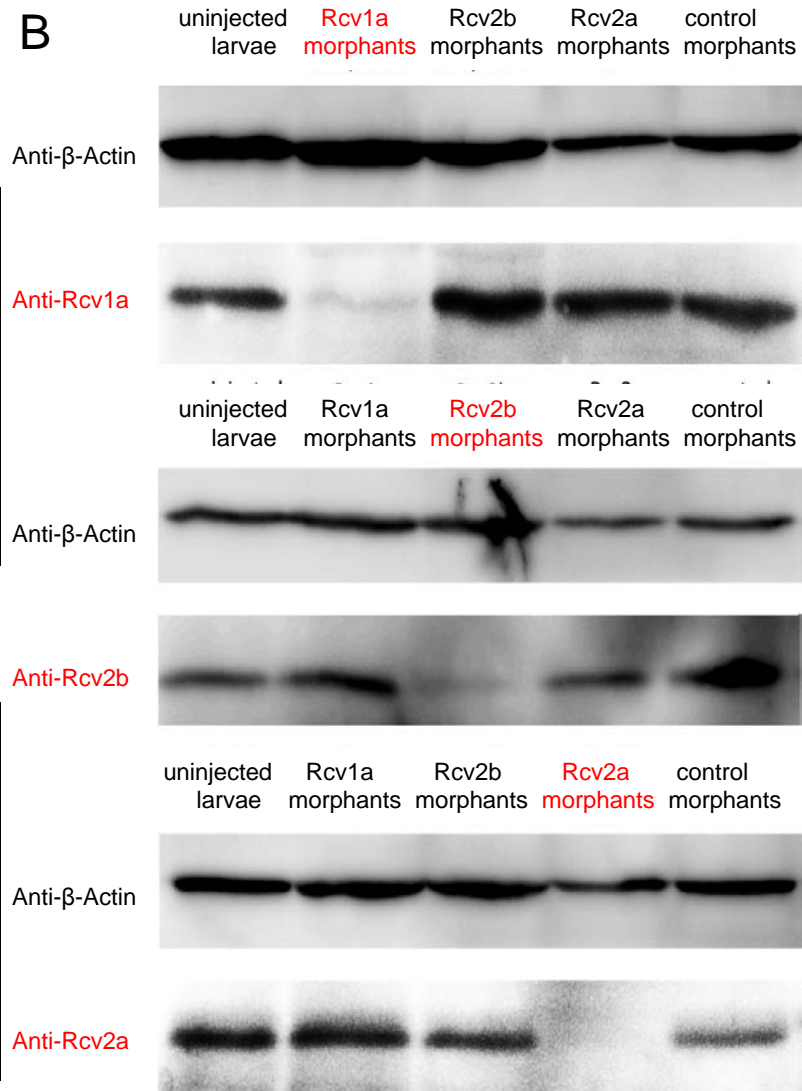

Supplement: Morpholino knockdown of Rcv1a, Rcv2a, and Rcv2b in 5dpf Larvae [file rsob150086supp5.pdf]

GRK7a/zpr1

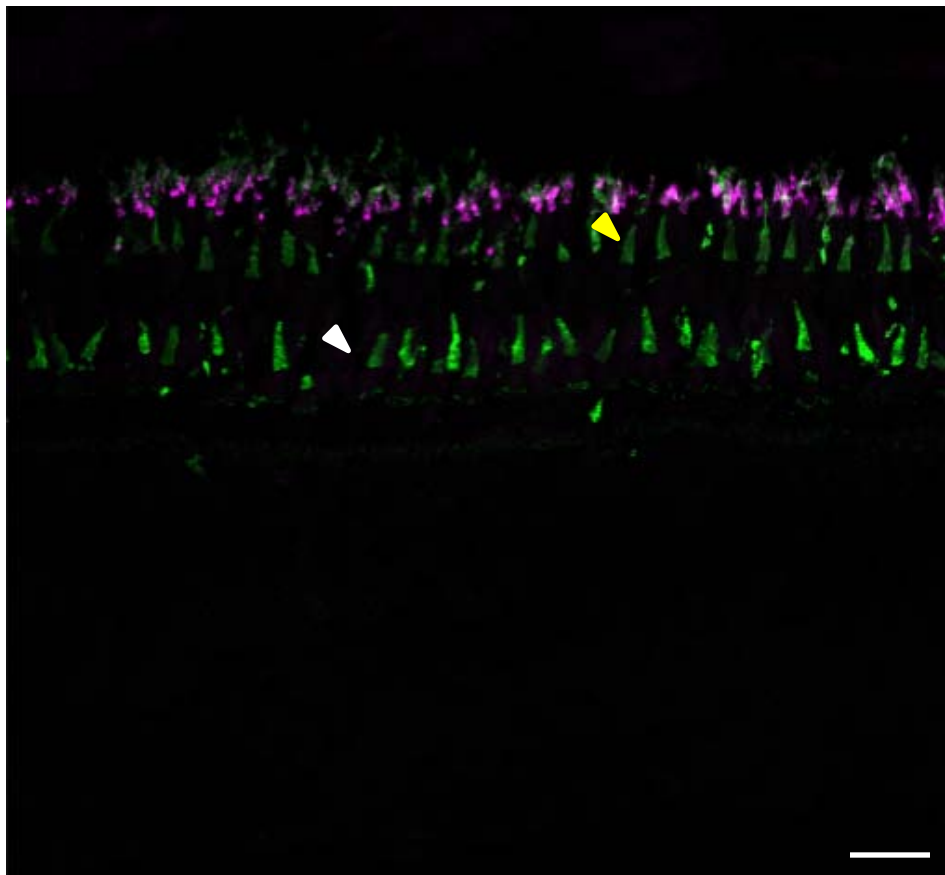

OS

ONL  
OPL

INL

Supplement: Grk7a expression in adult retina sections [file rsob150086supp6.pdf]
